# Prostaglandin pathways in equine myometrium regulations: endometrosis progression

**DOI:** 10.3389/fvets.2024.1479508

**Published:** 2024-12-05

**Authors:** Katarzyna K. Piotrowska-Tomala, Anna Z. Szóstek-Mioduchowska, Ewa M. Drzewiecka, Agnieszka W. Jonczyk, Anna Wójtowicz, Michał H. Wrobel, Graca Ferreira-Dias, Dariusz J. Skarzynski

**Affiliations:** ^1^Reproductive Immunology and Pathology, Institute of Animal Reproduction and Food Research Polish Academy of Science, Olsztyn, Poland; ^2^Gamete and Embryo Biology, Institute of Animal Reproduction and Food Research Polish Academy of Science, Olsztyn, Poland; ^3^Physiology and Toxicology, Institute of Animal Reproduction and Food Research Polish Academy of Science, Olsztyn, Poland; ^4^CIISA- Center for Interdisciplinary Research in Animal Health, Faculty of Veterinary Medicine, University of Lisbon, Lisbon, Portugal; ^5^AL4AnimalS-Associate Laboratory for Animal and Veterinary Sciences, Lisbon, Portugal

**Keywords:** prostaglandin E_2_, prostaglandin F_2α_, contractions, myometrium, mare, endometrosis

## Abstract

**Introduction:**

Prostaglandins (PG) are important regulators of the myometrial contractility in mammals. Endometrosis, a condition characterized by morphological changes in the equine endometrium, also affects endometrial secretory function. However, it remains unclear whether and how endometrosis affects myometrial function.

**Methods:**

This study investigated: (i) mRNA transcription of genes encoding specific enzymes responsible for PG synthesis, such as prostaglandin—endoperoxide synthase (*PTGS2*), PGE_2_ synthase (*PTGES*), PGF_2α_ synthase (*PTGFS*) and *PG receptors*: PGE_2_ receptors (*PTGER1- 4*), and PGF_2α_ receptor (*PTGFS*) in equine myometrium and, (ii) the effects of PGE_2_ and PGF_2α_ on myometrial contractile activity, during endometrosis in mares. The myometria used in experiments 1 and 2 were collected from mares in the mid-luteal (*n* = 23) and follicular (*n* = 20) phases of the estrous cycle, according to the histological classification of the endometrium (Kenney and Doig categories I, IIA, IIB, and III).

**Results:**

In experiment 1, changes in mRNA transcription of *PG synthase* or *PG receptors* in the myometrium during the course of endometrosis were determined using qPCR. During the mid-luteal phase, myometrial mRNA transcription of *PTGES* increased in mares with endometrial category IIB compared to category I. However, myometrial mRNA transcription of *PTGER1* decreased during the progression of endometrosis compared to category I. During the follicular phase, mRNA transcription of *PTGER1* and *PTGER2* increased in mares with endometrial categories III or IIA, respectively. In addition, mRNA transcription of *PTGFS* increased in mares with endometrium category IIA compared to category I. In experiment 2, the force of myometrial contractions was measured using an isometric concentration transducer. In the follicular phase, PGE_2_ decreased the force of contractions in mares with endometrial categories IIA, IIB, and III compared to the respective control groups. Prostaglandin F_2α_ increased the force of myometrial contractions in mares with category IIA endometrium, whereas it decreased in category IIB compared to the respective control groups.

**Discussion:**

We concluded that in the progression of endometrosis there are changes in the myometrial transcription of mRNA encoding *PG synthases* and *receptors*, particularly *PTGER1* and *PTGER2*. Mares with endometrosis had abnormal myometrial contractile responses to PG. These findings suggest that myometrial function may be compromised during the progression of endometrosis.

## Introduction

Endometrosis is a chronic degenerative condition that affects the uterus of mares, leading to significant economic losses in the equine industry (1–3). The main hallmarks of endometrosis are fibrotic changes in the endometrium, collagen formation around the endometrial glands and/or in the stroma, cystic dilation, as well as dilation of lymphatic vessels (4–8). There have been several studies on the pathogenesis of endometrosis and the secretory function of the endometrium during this condition including secretion and actions of prostaglandins (PG) (8–13). However, little is currently known about the myometrial changes that occur in endometrosis. Hanada et al. (14) observed histopathological changes in equine myometrium in relation to the stage of endometrosis. These changes include the degeneration of smooth muscle cells and lymphatic lacunae in the tissue's vascular layer.

The myometrium's primary function is its contractile activity, responsible for transporting sperm or gametes in the equine reproductive tract, and involved in mating, parturition, and uterine involution (15–19). Insufficient myometrial activity can cause uterine clearance issues that are associated with post-breeding endometritis (20, 21). This can lead to infertility and implantation failure in mares (22). Moreover, there is evidence that endometrosis and endometritis are linked processes (23, 24).

Prostaglandins regulate myometrial contractile activity in mares throughout the estrous cycle (25–28) and during the time period of embryo mobility (27, 29–33). Equine myometrium has recently been shown to be an additional source of PG (34).

Prostaglandin synthesis starts with converting arachidonic acid (AA) into prostaglandin H_2_ (PGH_2_) by prostaglandin-endoperoxide synthases (PTGS2). Prostaglandin F_2α_ synthase (PTGFS) and PGE_2_ synthase (PTGES) catalyze the production of prostaglandin F_2α_ (PGF_2α_) or prostaglandin E_2_ (PGE_2_), respectively (35). Prostaglandins interact with membrane-bound receptors in the equine endometrium (36). Prostaglandin F_2α_ receptor (PTGFR) binds PGF_2α_, while PGE_2_ receptor (PTGER) has four subtypes: PTGER1, PTGER2, PTGER3, and PTGER4 (36). According to Wanggren et al. (37), PTGER1 and PTGER3 induce smooth muscle contraction, whereas PTGER2 and PTGER4 induce smooth muscle relaxation. Previous studies highlighted mRNA transcription changes of *PG synthase* and PG concentration in equine endometrium across endometrosis stages (9, 10, 13). However, the expression status of PG synthases and receptors in myometrium during endometrosis remains unexplored.

We hypothesize that mRNA transcription of *PG synthase* and *PG receptor* in equine myometrium is disrupted during endometrosis establishment. Additionally, this study aims to evaluate the differences in myometrial contractile activity mediated by PGE_2_ or PGF_2α_ in mares with endometrosis during the mid-luteal and follicular phases of the estrous cycle.

## Materials and methods

### Animals and material collection

Uterine samples (*n* = 43) were collected post-mortem from Polish cold-blood mares with ovarian cyclicity, weighing 500 ±100 kg and ranging from 2 to 20 years of age during the breeding season (between April and June) at a local abattoir (Rawicz, Poland). The mares were confirmed to be clinically healthy by an official government Veterinary inspector, and by referral to historical health records for each animal. The animals were slaughtered as part of routine meat production protocols at a local abattoir. The slaughter process was conducted in accordance with European legislation (EFSA, AHAW/04-027) to ensure the elimination of pain and suffering. All material collection procedures were approved by The Local Ethics Committee for Experiments on Animals in Olsztyn, Poland (Agreement No. 51/2011). The phase of the estrous cycle was determined based on progesterone (P_4_) analysis and macroscopic observation of the ovaries (38). Hence, the mid-luteal phase is characterized by a well-developed corpus luteum (CL) associated with 15–20 mm follicles. In contrast, the follicular phase is characterized by the absence of an active CL, the presence of follicles larger than 35 mm in diameter.

Myometrial samples were taken from all uteri from which the endometrium was obtained. The endometrium was washed with cold sterile RNAse-free saline solution and placed into 4% buffered paraformaldehyde (POCH, Gliwice, Poland, #432173111) for histological analysis after hematoxylin-eosin staining (38). The endometria were classified retrospectively into categories I, IIA, IIB, or III based on the Kenney and Doig classification (1), which considers fibrosis, inflammatory infiltrates, and the degree of dilatation of endometrial glands and lymphatic vessels.

Myometrium was excised from the endometrium and perimetrium of the uterine horns that were ipsilateral to the CL (mid-luteal phase of the estrous cycle) or the growing follicle (follicular phase of the estrous cycle). The excised myometrium was then washed with cold, sterile, RNAse-free saline solution and stored in RNAlater (#AM7021; Invitrogen) at −80°C for further qPCR analyses (experiment 1). To measure myometrial activity in experiment 2, a 3–4 mm wide and 6–7 mm long myometrium was excised and cut in the direction of the longitudinal muscle. Each myometrial strip was washed with cold sterile RNAase-free saline solution and placed into 2 mL of aerated physiologic salt solution (PSS, pH 7.4) at 4°C with 95% air and 5% CO_2_ until measuring myometrial contractility (39). The measurement of myometrial contractility was carried out before histopathological categorization of the endometrium, according to Kenney and Doig (1), to obtain the appropriate number of samples in each experimental group.

Myometrial samples were linked to the endometrium that was previously assigned according to Kenney and Doig endometrial histopathological grading (1), in conjunction with an assessment of the phase of the estrous cycle. The same myometrial were used for both experiment 1 and experiment 2.

### Experimental procedures

Experiment 1. Myometrial PG synthases and PG receptors mRNA transcription at different stages of mare's endometrosis.

Myometrial samples were retrospectively assigned to endometrial categories I, IIA, IIB, or III according to the Kenney and Doig classification system (1). Myometria from the mid-luteal phase (*n* = 6 for category I, *n* = 6 for category IIA, *n* = 5 for category IIB, *n* = 6 for category III); and the follicular phase (*n* = 5 for category I, *n* = 6 for category IIA, *n* = 5 for category IIB, *n* = 4 for category III) of the estrous cycle were used for the determination of *PTGS2, PTGES, PTGFS, PTGER1, PTGER2, PTGER3, PTGER4*, and *PTGFR* mRNA transcription at different stages of mare endometrosis. Myometrial samples were taken from the same uteri for experiments 1 and 2.

Experiment 2. The effects of PGE_2_ and PGF_2α_ on myometrial contractile activity from different categories of mare endometrium.

Myometrial samples used in this experiment were from the same uteri as those used in experiment 1. Myometrial tissues from the mid-luteal (*n* = 6 for category I, *n* = 6 for category IIA, *n* = 5 for category IIB, *n* = 6 for category III) and follicular phases of the estrous cycle (*n* = 5 for category I, *n* = 6 for category IIA, *n* = 5 for category IIB, *n* = 4 for category III) were utilized to determine the effect of PGE_2_ and PGF_2α_ on myometrial contractile activity at different categories of mare endometrium. Myometrial strips were treated with PGE_2_ (Cayman Chemical, Ann Arbor, MI, USA #14010) or PGF_2α_ (Cayman Chemical, Ann Arbor, MI, USA #16010) at increasing doses of 10^−8^M, 10^−7^M, and 10^−6^M for 5 min. The concentrations of PGE_2_ and PGF_2α_ were chosen based on a preliminary study carried out by us, and previous studies by Word et al. (40), Rigby et al. (28), and Chioss et al. (41). The force of myometrial contractions was measured using an isometric concentration transducer (HSE Type 372, HSE Schuler Organ bath apparatus, March-Hugstetten, Germany).

### RNA extraction, cDNA synthesis, and real-time qPCR

Total RNA was extracted from myometrial tissues using the TRI Reagent^®^ (T9424-200 ML, Sigma Aldrich, Germany) following the manufacturer's instructions. RNA content and purity were evaluated using a NanoDrop 1000 Spectrophotometer (Thermo Fisher Scientific, ND-1000, Wilmington, DE, USA). The A260/280 absorbance ratio for all samples was ~2.0, and the 260/230 absorbance ratio ranged between 1.8 and 2.0. The RNA was reverse transcribed into cDNA using the Reverse Transcription Kit (Qiagen, Hilden, Germany, #205311) at a concentration of 1.5 μg.

Real-time PCR was performed using TaqMan Universal Master Mix II (4440049; Applied Biosystems, Foster City, CA, USA) on a Viia7 system (Applied Biosystems, Waltham, Massachusetts, USA) in 384-well plates. All samples were run in duplicates. To measure mRNA transcription of analyzed genes, i.e., *PTGS2* (cat. no. Ec03467558_m1), *PTGES* (cat. no. Ec04321097_s1), *PTGFS* (cat.no. Ec03468273_m1), *PTGER1* (cat.no. Ec07038494_m1), *PTGER2* (cat. no. Ec03469468_m1), *PTGER3* (cat.no. Ec07094974_m1), *PTGER4* (cat.no. Ec03397708_g1), *PTGFR* (cat. no. Ec03467807_m1) in relation to reference genes, i.e., ubiquitin conjugating enzyme E2 (*UBE2B*) (cat. no. Ec07038512), ribosomal protein L32 (*RPL32*) (cat. no. Ec06951800_m1), and succinate dehydrogenase complex flavoprotein subunit A (*SDHA*) (cat. no. Ec03470487_m1), Single Tube TaqMan Gene Expression Assays (Life Technologies Thermo Fisher Scientific) were used. The reaction mixture for the qPCR assay consisted of 5 μL TaqMan Universal PCR Master Mix, 0.5 μL TaqMan probe, 3 μL cDNA (2.5 ng) and 1.5 μL nuclease-free water to a final volume of 10 μL. As a negative control, nuclease-free water was used instead of template cDNA. cDNA amplification was performed under the following conditions: initial denaturation for 10 min at 95°C, followed by 40 cycles of 15 s at 95°C and 1 min at 60°C. Data were analyzed using the method described previously by Zhao and Fernald (42) using the equation R0 = 1/(1 + E)Ct, where E is the average gene efficiency and Ct is the number of cycles at the threshold. Relative gene expression was calculated as R0 target gene/ R0 reference gene and expressed in arbitrary units. The stability of used reference genes was tested and confirmed using NormFinder software (43).

### Measurement of myometrial contractility

**Figure 1:**
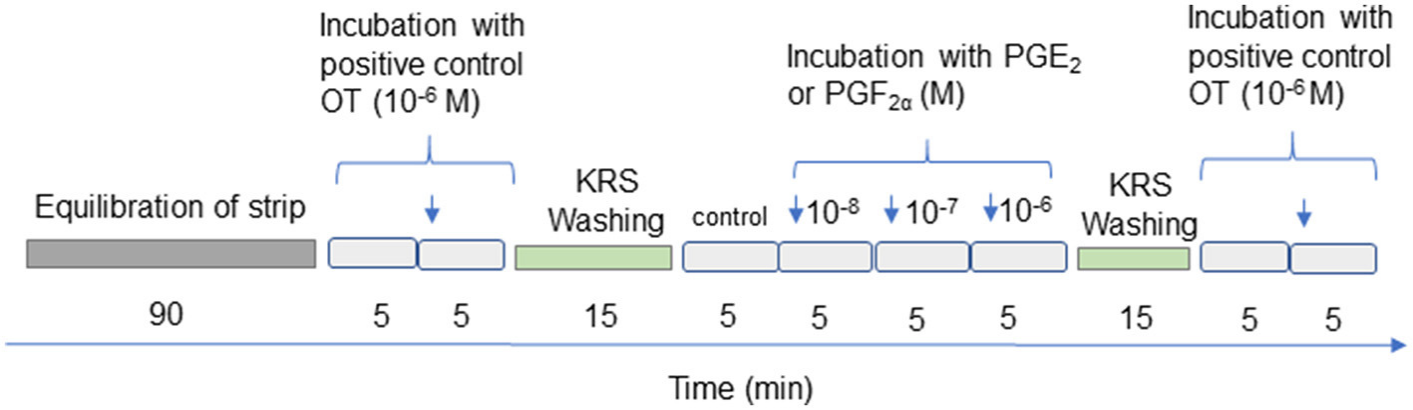
Diagram showing treatment of the myometrial strips. OT, oxytocin; PGE_2_, prostaglandin E_2_, PGF_2α_, prostaglandin F_2α_. Concentrations of the examined substances are expressed in moles (M).

Figure 1 depicts the experimental protocol for measuring uterine contractility. Each myometrial strip was individually attached to the base of the chambers using the HSE Schuler Organ bath apparatus. The strip was then tied to the isometric contraction transducer HSE Type 372, using a stationary hook and surgical silk. The chambers contained Krebs-Ringer's solution (KRS) with a pH of 7.4 and a volume of 10 mL. The solution was composed of NaCl (120.3 mM), KCl (5.9 mM), CaCl_2_ (2.5 mM), MgCl_2_ (1.2 mM), NaH_2_PO_4_ (1.2 mM), NaHCO_3_ (15.5 mM), and glucose (11.5 mM), as described by Wrobel et al. (39). The baths were constantly oxygenated with a mixture of 95% O_2_ and 5% CO_2_ and were maintained at a temperature of 38.5°C. All preparations were allowed to equilibrate for 90 min. During the pre-incubation period, we observed spontaneous and regular contractions of the myometrial strips, which we refer to as the stabilization period. The viability and usefulness of smooth muscle tissue isometric contractions were measured every 2 s for 5 min before (basal contractions) and after the application of oxytocin (OT), as previously described (39, 44). To serve as a positive control, we used OT treatment at a concentration of 1 μM (Sigma Aldrich, #04375). Within 5 min, OT clearly stimulated myometrial contraction, which was then stabilized (data not shown). To reassess tissue functionality, the same dose of OT was used as before. The tissues in the chambers were rinsed three times using KRS after OT stimulation. Only results where the difference in response to OT stimulation (1 μM) at the beginning and end of the study was < 20% were included in the statistical analysis.

Myometrial contractility was measured every 2 s for 5 min in response to PGE_2_ or PGF_2α_ at doses of 10^−8^M, 10^−7^M, and 10^−6^M.

### Statistical analysis

For each statistical analysis, a Gaussian distribution was tested using the D'Agostino & Pearson normality test (GraphPad Prism 10.2.1 software; GraphPad, San Diego, CA, USA). In experiment 1, a two-way ANOVA followed by Sidak‘s multiple comparison test (GraphPad Prism) was used. Gene expression differences in the myometrium of mares with Kenney and Doig categories I, IIA, IIB, and III endometria were assessed between the mid-luteal and follicular phases of the estrous cycle (different superscript letters) and between categories IIA, IIB, and III compared with category I in each phase of the estrous cycle (asterisk). In experiment 2, we conducted a statistical analysis of the force of myometrial contraction using two-way ANOVA for repeated measures, followed by the Sidak‘s multiple comparison test (GraphPad). The mean contraction force values (mN) ± SEM were calculated based on measurements collected every 2 s over a 5-min period. Results were considered significant at *P* < 0.05.

## Results

Experiment 1. The determination of PG synthases and PG receptors mRNA transcription in equine myometrium at different stages of endometrosis.

**Figure 2:**
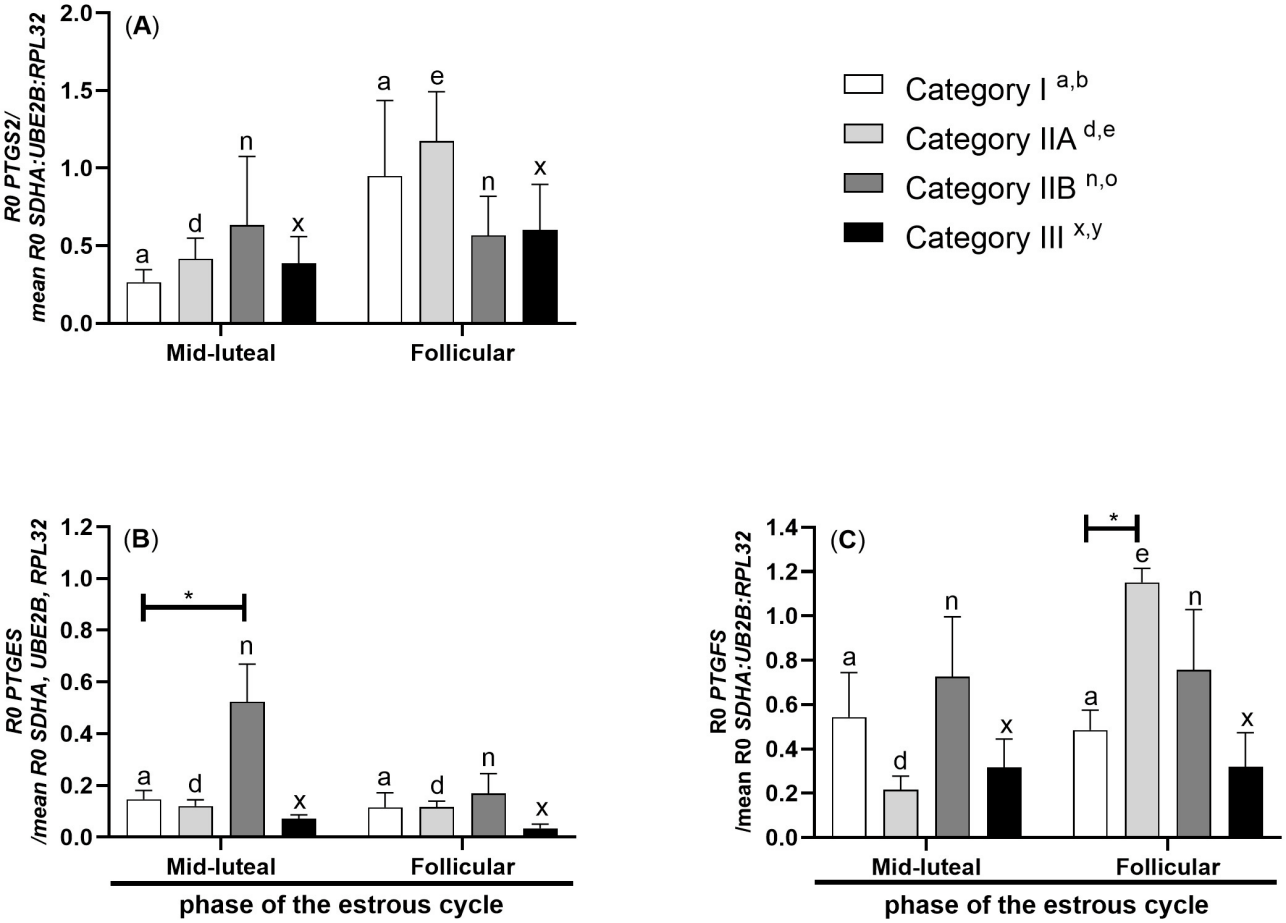
Myometrial mRNA transcription of *PG synthases* in mares with endometrosis at different stages of the estrous cycle. mRNA transcription of **(A)** *PTGS2*, **(B)** *PTGES*, and **(C)** *PTGFS* in mares with category I (*n* = 6), IIA (*n* = 6), IIB (*n* = 5), and III (*n* = 6) endometrium during the mid-luteal phase or with category I (*n* =5), IIA (*n* = 6), IIB (*n* = 5), and III (*n* = 4) endometrium during the follicular phase of the estrous cycle. Bars represent mean ± SEM. The superscript indicates statistical differences in myometrium in categories I^a, b^, IIA^d, e^, IIB^n, *o*^, and III^x, y^ between mid-luteal and follicular phases. The asterisk indicates statistical differences in mRNA transcription of *PG synthase* in myometria compared to category I, within the mid-luteal or follicular phase of the estrous cycle (*P < 0.05).

In mares with endometrium category IIA, *PTGS2* mRNA transcription was lower in the myometrium during the mid-luteal stage compared to the follicular phase of the estrous cycle (*P* < 0.05; Figure 2A).

mRNA transcription of *PTGES* was found to be upregulated in the myometrium of mares with endometrium category IIB during the mid-luteal stage of the estrous cycle compared to mares with endometrium category I (*P* < 0.05; Figure 2B).

In mares with endometrium category IIA, *PTGFS* mRNA transcription was lowered in the myometrium during the mid-luteal phase of the estrous cycle compared to the myometrium during the follicular phase of the estrous cycle (*P* < 0.05; Figure 2C). mRNA transcription of *PTGFS* increased in the myometrium of mares with endometrium category IIA during the follicular phase of the estrous cycle compared to mares with endometrium category I (*P* < 0.001; Figure 2C).

**Figure 3:**
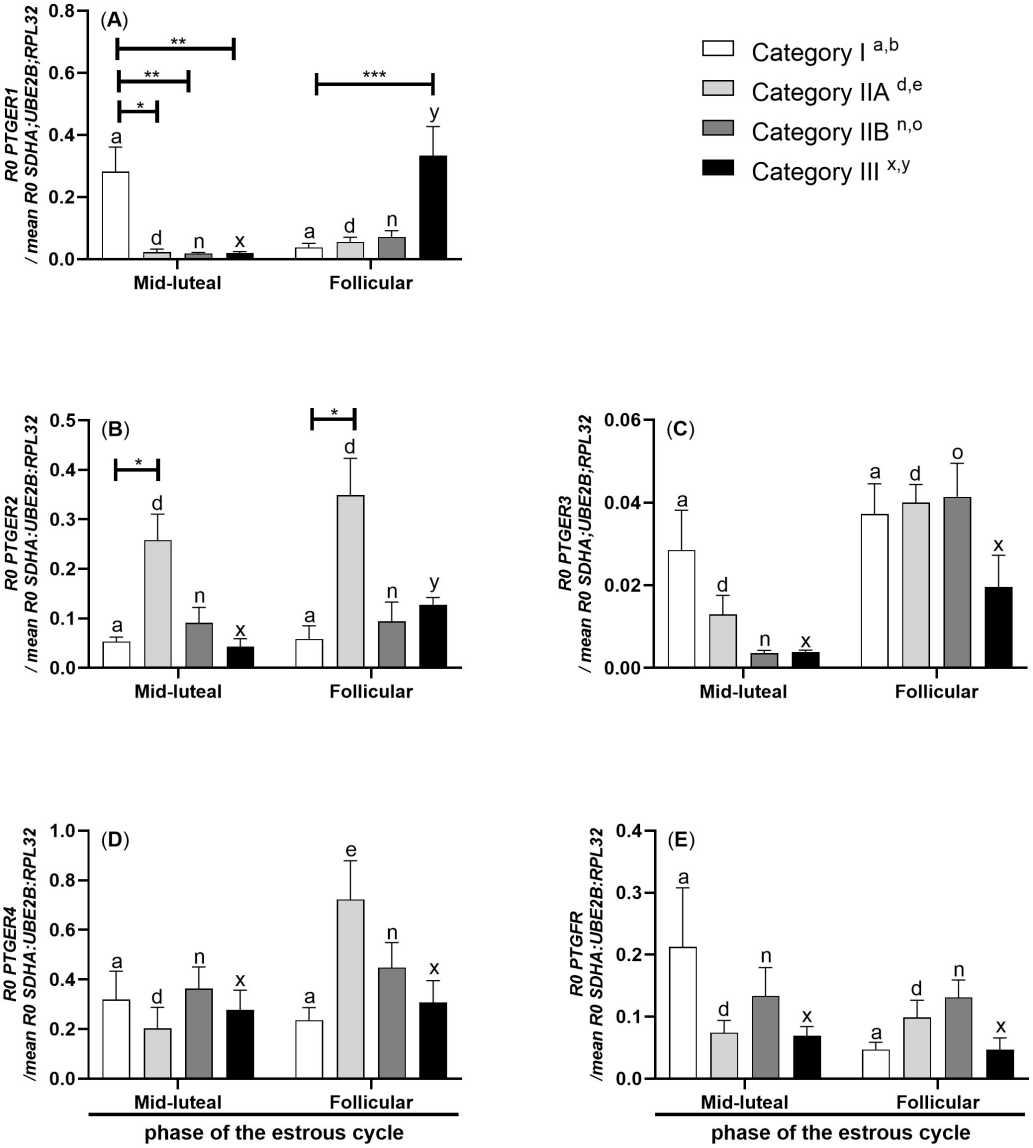
Myometrial mRNA transcription of *PG receptors* in mares with endometrosis at different stages of the estrous cycle. mRNA transcription of **(A)** *PTGER1*, **(B)** *PTGER2*, **(C)** *PTGER3*, **(D)** *PTGER4*, and **(E)** *PTGFR* in mares with category I (*n* = 6), IIA (*n* = 6), IIB (*n* = 5), and III (*n* = 6) endometrium during the mid-luteal or with category I (*n* =5), IIA (*n* = 6), IIB (*n* = 5), and III (*n* = 4) endometrium during the follicular phase of the estrous cycle. Bars represent mean ± SEM. The superscript indicates statistical differences in myometrium in categories I^a, b^, IIA^d, e^, IIB^n, *o*^, and III^x, y^ between mid-luteal and follicular phases. The asterisk indicates statistical differences in mRNA transcription of *PG receptors* in myometria compared to category I, within the mid-luteal or follicular phase of the estrous cycle (*P < 0.05; **P < 0.01; ***P < 0.001).

In mares with endometrium category III, *PTGER1* mRNA transcription was lowered in the myometrium during the mid-luteal phase of the estrous cycle compared to the follicular phase (*P* < 0.05; Figure 3A). mRNA transcription of *PTGER1* was decreased in the myometrium of mares with endometrium categories IIA, IIB, and III in the mid-luteal phase of the estrous cycle compared to the myometrium of mares with endometrium category I (*P* < 0.05; *P* < 0.01; *P* < 0.01, respectively; Figure 3A). While during the follicular phase, its mRNA transcription was increased in the myometrium of mares with endometrium category III compared to the myometrium of mares with endometrium category I (*P* < 0.001; Figure 3A).

In mares with endometrium category III, *PTGER2* mRNA transcription was lowered in the myometrium during the mid-luteal phase of the estrous cycle compared to the follicular phase (*P* < 0.05; Figure 3B). mRNA transcription of *PTGER2* was increased in the myometrium of mares with endometrium category IIA in both phases of the estrous cycle compared to myometrium of mares with endometrium category I (*P* < 0.05; Figure 3B).

In mares with endometrium category IIB, *PTGER3* mRNA transcription was lowered in the myometrium during the mid-luteal phase of the estrous cycle compared to the myometrium during the follicular phase (*P* < 0.01; Figure 3C).

In mares with endometrium category IIA, *PTGER4* mRNA transcription was lowered in the myometrium during the mid-luteal phase compared to the follicular phase of the estrous cycle (*P* < 0.05; Figure 3D).

There were no statistically significant differences in the mRNA transcription of *PTGFR* in the mare myometrium between different stages of endometrosis and phases of the estrous cycle (*P* > 0.05; Figure 3E).

Experiment 2. The effect of prostaglandin E_2_ and prostaglandin F_2α_ on myometrial contractile activity from different categories of mare's endometrium.

**Figure 4:**
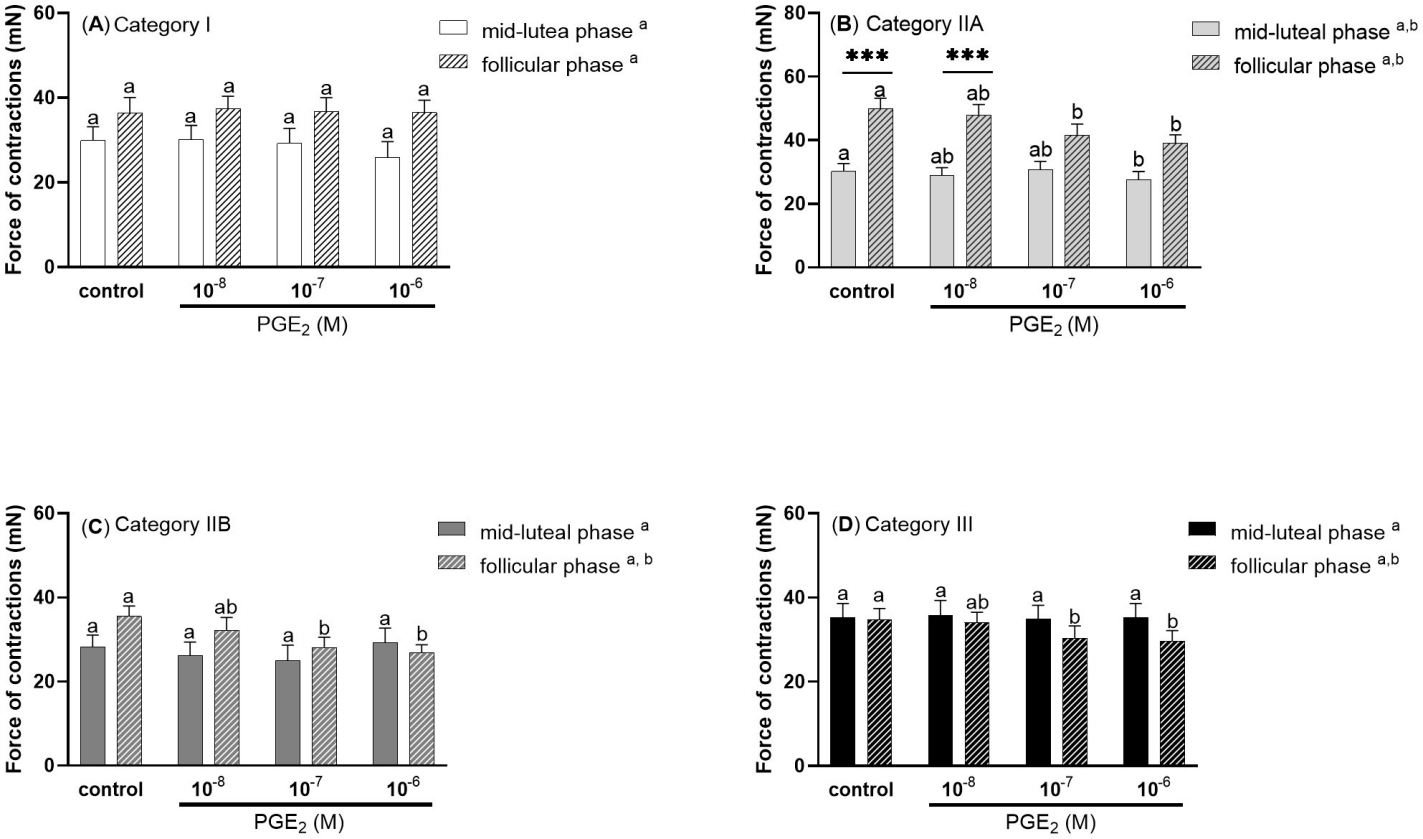
The force (mean ± SEM) of myometrial contraction of control and PGE_2_-stimulated (10^−8^M, 10^−7^M, 10^−6^M; patterned bars) strips from mares with categories of endometrium: **(A)** I, **(B)** IIA, **(C)** IIB, and **(D)** III collected at mid-luteal phase (*n* = 6 for category I, *n* = 6 for category IIA, *n* = 5 for category IIB, *n* = 6 for category III) or follicular phase (*n* = 5 for category I, *n* = 6 for category IIA, *n* = 5 for category IIB, *n* = 4 for category III) of the estrous cycle. The asterisk indicates statistical differences in the myometrial contractile force of the PGE_2_-stimulated myometrial strip compared to the respective control group (***P < 0.001) in each category of endometrium during mid -luteal or follicular phase of the estrous cycle. Different superscript letters (^a, b^) indicate statistical significance in the force of the myometrial contraction within control groups or PGE_2_ -stimulated myometrial strips between mid-luteal phase vs. follicular phase of the estrous cycle (*P* < 0.05).

There were no statistically significant differences in the force of contractions of control and PGE2 stimulated stripes from mares with category I endometrium (*P* > 0.05; Figure 4A). Prostaglandin E_2_ at a dose of 10^−6^M was found to downregulate the force of myometrial contractions in mares with endometrium category IIA during the mid-luteal phase of the estrous cycle, compared with the control group (*P* < 0.05; Figure 4B). While, during the follicular phase of the estrous cycle, PGE_2_ at a dose of 10^−7^ and 10^−6^M decreased the force of contractions in mares with endometrium category IIA (*P* < 0.05; Figure 4B), IIB (*P* < 0.05, Figure 4C), and III (*P* < 0.05, Figure 4D), compared with respective control groups. Moreover, in mares with category IIA endometrium, the force of myometrial contraction was significantly higher in both the control and PGE_2_ (10^−8^M) treated groups during the follicular phase compared to the respective groups in the mid-luteal phase of the estrous cycle (*P* < 0.001; Figure 4B).

**Figure 5:**
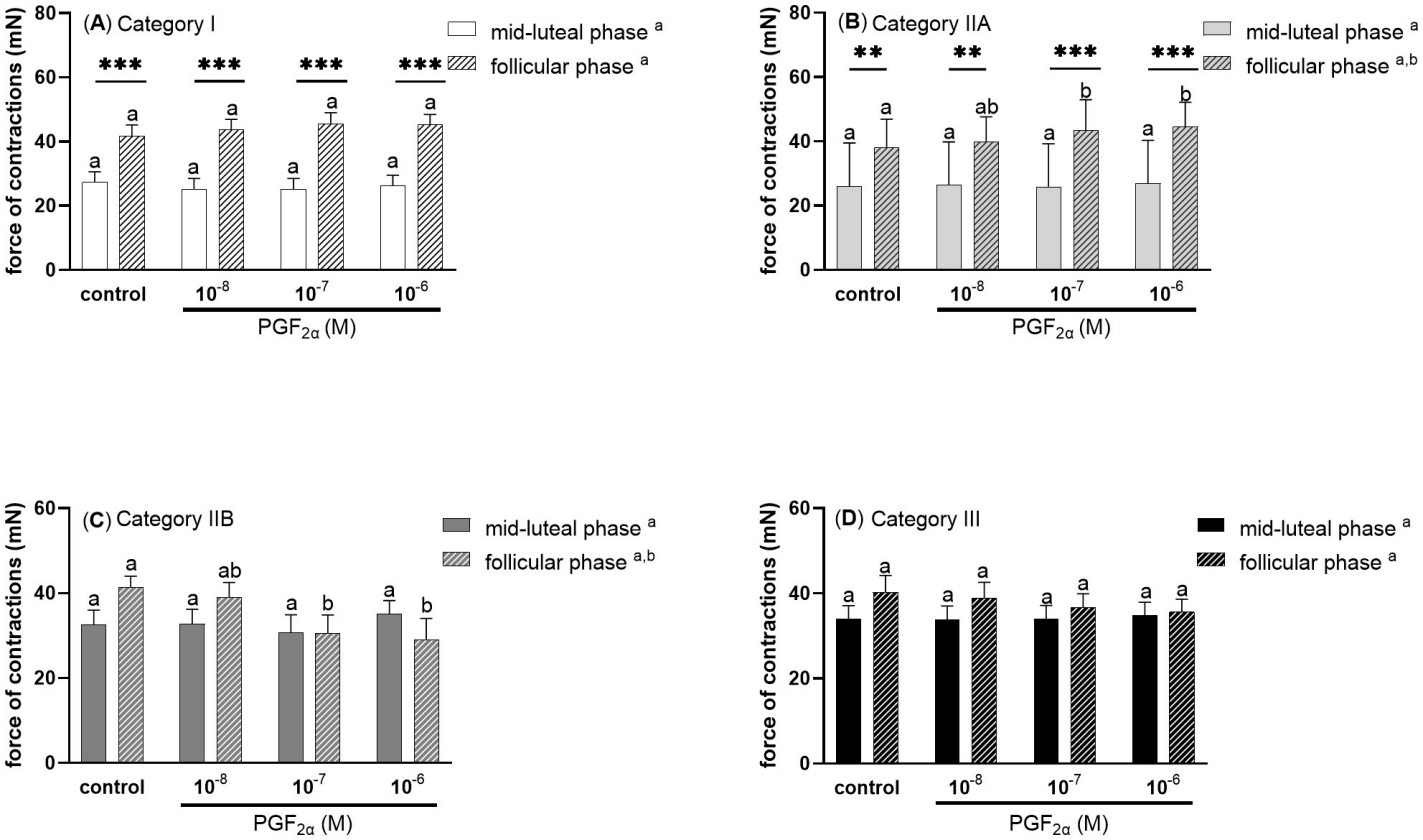
The force (mean ± SEM) of myometrial contraction of control and PGF_2α_-stimulated (10^−8^M, 10^−7^M, 10^−6^M; patterned bars) strips from mares with categories of endometrium: **(A)** I, **(B)** IIA, **(C)** IIB, and **(D)** III collected at mid-luteal phase (*n* = 6 for category I, *n* = 6 for category IIA, *n* = 5 for category IIB, *n* = 6 for category III) or follicular phase (*n* = 5 for category I, *n* = 6 for category IIA, *n* = 5 for category IIB, *n* = 4 for category III) of the estrous cycle. The asterisk indicates statistical differences in the myometrial contractile force of PGF_2α_-stimulated myometrial strip compared to the respective control group (**P < 0.01, ***P < 0.001) in each category of endometrium during mid -luteal or follicular phase of the estrous cycle. Different superscript letters (^a, b^) indicate statistical significance in the force of the myometrial contraction within control groups or PGF_2α_ -stimulated myometrial strips between mid-luteal phase vs. follicular phase of the estrous cycle (*P* < 0.05).

Prostaglandin F_2α_ at doses 10^−7^ and 10^−6^M upregulated the force of myometrial contractions in mares with endometrium category IIA, in the follicular phase of the estrous cycle (*P* < 0.05; Figure 5B), while it decreased the force of myometrial contractions in mares with category IIB endometrium (*P* < 0.05; Figure 5C), compared with respective controls. In mares with category I or IIA endometrium, the force of myometrial contraction was significantly higher in the control and PGF_2α_ (10^−8^M, 10^−7^M, 10^−6^M) treated groups during the follicular phase compare to the respective groups in the mid-luteal phase of the estrous cycle (*P* < 0.01; Figures 5A, B). There were no statistically significant differences in the force of contractions of control and PGE2α stimulated stripes from mares with category III endometrium (*P* > 0.05; Figure 5D).

## Discussion

Histological changes in the myometrium of mares with endometrosis and reduced myometrial contractile activity (14, 45) may contribute to subfertility or infertility observed in mares with this condition (1, 8, 46). To better understand the underlying mechanisms of myometrial contractile dysfunction associated with equine endometrosis, it is essential to investigate the regulatory pathways of PG secretion and action in the myometrium, as well as the effect of PG on myometrial contractile activity.

Therefore, in this study, we clarified the mRNA transcription of *PG synthases* and *PG receptors* in the myometrium of mares with different endometrial categories, classified according to the Kenney and Doig system. Additionally, we examined the myometrial response to PG across different endometrial categories. To the best of our knowledge, this study is the first to identify disruptions in the PG synthesis pathway in the myometrium of mares during progression of endometrosis. These alterations in *PG synthase* mRNA transcription may affect the local uterine microenvironment, potentially impairing myometrial function during the estrous cycle.

Our results revealed that myometrial mRNA transcription of PG receptors *PTGER1, PTGER2, PTGER3*, and *PTGER4* remained unchanged during the mid-luteal and follicular phases of the estrous cycle in mares with a category I endometrium. This is consistent with the findings of Silva et al. (47), who also reported no significant differences in *PTGER2* and *PTGER4* mRNA transcription between preovulatory estrus, late diestrus, and pregnancy, in both equine endometrium and the myometrium. In our study, in mares during progression of endometrosis, notable alternations in *PGE*_2_ *receptor* mRNA transcription in the myometrium was observed. Specifically, mRNA transcription of *PTGER2* was significantly elevated in the myometrium of mares with category IIA endometrium during both phases of the estrous cycle compared to mares with category I endometrium. While, myometrial *PTGER4* transcription decreased in mare with category IIA endometrium during the mid-luteal phase of the estrous cycle. Additionally, mRNA transcription of *PTGER1* was significantly altered in the myometrium of mares with endometrial categories IIA, IIB and III compared to mares with category I endometrium, particularly during the mid-luteal phase of the estrous cycle. During the follicular phase, myometrial mRNA transcription of *PTGER1* increased in mares with category III endometrium compared to mares with category I endometrium. These findings suggest that receptor- specific changes in transcription are associated with the progression of endometrosis in mares. Interestingly, we found that myometrial mRNA transcription of *PGFR* remained unchanged across all endometrosis categories. However, alterations in myometrial contractility in response to PGF_2α_ were observed during the follicular phase of the estrous cycle, indicating that activity of the myometrium may not be solely attributable to changes in the expression of PG receptors alone. This suggests that additional factors, such as alterations in molecular signaling pathways activated by PG, may contribute to the observed contractile dysfunction. The limitation of this study is that post-transcriptional analysis of PG synthase and its receptors was not performed. The mRNA levels do not always correlate directly with protein expression. Therefore, it can be postulated that discrepancies between the transcription level and the myometrial contraction patterns may be explained by different posttranscriptional changes in their expression within the myometrium during the progression of endometrosis. This should be the direction of future studies.

The role of the myometrium is mainly related to its motility and its proper regulation is essential for optimal reproductive performance in mare (18). In present study, reduced contractile activity was observed in response to PGE_2_ in myometrium of mares with category IIA, IIB, and III endometria compared to respective control groups, particularly during the follicular phase of the estrous cycle. Myometrium of mares with category IIB endometrium also exhibited reduced contractility in response to PGF_2α_ despite unchanged myometrial *PGFR* mRNA transcription. These findings suggest that myometrial contractile activity during endometrosis compared to healthy mare is reduced in response to PG. In veterinary practice PGF_2α_ or oxytocin (OT) is commonly administered post-breeding to stimulate uterine contractions and clear intraluminal fluid. Mares with delayed uterine clearance or chronic endometritis exhibit diminished responsiveness to PGF_2α_ relative to healthy mares (14, 28, 48, 49). This diminished response has been linked to a contractile defect in the myometrium, particularly in older mares and those with advanced endometrosis, who are prone to persistent breeding-induced endometritis (50). We can assume that the mares in our study with category IIB endometrium had a reduced myometrial contractile response to PGF_2α_, suggesting that traditional therapies may be less effective in these cases. This reduced myometrial contractile function could contribute to poor uterine clearance and persistence of infections, negatively impacting fertility. The age range of the mares used in our study was between 2 and 20 years. The wide age range of the animals was chosen to provide a cross-sectional view of endometrial fibrosis at different stages of its progression. As noted by Ebert et al. (51), endometrosis tends to increase with age, affecting 32% of mares under 5 years of age and 93% of mares over 20 years of age. By including mares across this wide age range, our study aimed to capture different stages of the condition. The progression of endometrosis may also be influenced by other age-related changes in the myometrium. Studies in other species have shown that advanced age is associated with increased collagen deposition in the endometrium and myometrium, leading to impaired uterine function (52, 53). In mares, older age and repeated uterine infections can result in endometrosis, which in turn contributes to subfertility or reproductive failure (8, 24, 54).

Beyond PG receptor expression, other molecular mechanisms may contribute to the myometrial contractile dysfunction observed in endometrosis. Rigby et al. (28) suggested that age-related changes in receptor-second messenger signaling pathways, particularly those downstream of intracellular Ca^2+^, may affect myometrial contractility. Indeed, smooth muscle contraction is regulated by several cellular mechanisms, including Ca^2+^/calmodulin-dependent myosin light chain kinase, Ca^2+^-independent regulatory light chain phosphorylation, myosin phosphatase inhibition, and actin filament-associated proteins (55–57). Moreover, changes in uterine blood flow, vascular structure, nerve supply, and increased collagen deposition may also play significant roles in reducing the tension-generating capacity of myometrial cells (4, 53, 58).

Examination of PG signaling pathways in the equine myometrium during reproductive pathologies could provide further insights into the broader roles of PG in reproductive disorders. Previous studies have shown that inflammation disrupts PG signaling in the equine uterus during endometritis (24, 59). In pregnancy-related conditions such as placentitis, inflammatory responses significantly alter PG signaling and contraction-associated pathways, such as OXTR, PTGS2 and connexin-43 (Cx43), affecting pregnancy outcomes (60, 61). Our recent study has shown that the myometrial mRNA transcription of OXTR remained unchanged between the Kenney and Doig endometrial categories and the phases of the estrous cycle (62). Therefore, these results suggest that the reduced myometrial activity is not related to reduced OXTR expression, but may be due to other processes mentioned above, such as age-related changes in receptor-second messenger signaling mechanisms downstream of intracellular Ca^2+^ release, as suggested by Rigby et al. (28). Given the importance of PG signaling in these pathological conditions, potential alterations in Cx43 expression could impair myometrial contractility in mares with endometrosis. Connexin-43 is essential in the myometrium, where it forms gap junctions that facilitate coordinated uterine contractions, a process important for effective labor (63). However, further study is necessary to clarify the molecular mechanisms contributing to myometrial dysfunction in mares with endometrosis.

In conclusion, our study demonstrates that myometrial contractile activity is altered in mares with endometrosis, in response to PG during the mid- luteal and follicular phases of the estrous cycle. Changes in myometrial mRNA transcription of *PG synthases* and *receptors*, such as *PTGER1* and *PTGER2*, were observed during endometrosis progression in mares. However, these alterations alone do not fully explain the disrupted myometrial contractile activity. The dysfunction likely involves complex interactions between multiple signaling pathways activated by PG in both the endometrium and myometrium. In addition, changes in the structure of the myometrium observed in mares during endometrosis may be related to the reduced tension-generating capacity of myometrial cells. Further research is needed to fully elucidate the molecular mechanisms underlying myometrial dysfunction in endometrosis and to identify potential therapeutic targets.

## Data Availability

The original contributions presented in the study are included in the article/supplementary material, further inquiries can be directed to the corresponding author/s.
